# Spatial distribution of Mycobacterium *Tuberculosis* in metropolitan Harare, Zimbabwe

**DOI:** 10.1371/journal.pone.0231637

**Published:** 2020-04-21

**Authors:** Joconiah Chirenda, Isaiah Gwitira, Robin M. Warren, Samantha L. Sampson, Amon Murwira, Collen Masimirembwa, Kudzanai M. Mateveke, Cremence Duri, Prosper Chonzi, Simbarashe Rusakaniko, Elizabeth M. Streicher

**Affiliations:** 1 Department of Community Medicine, College of Health Sciences, University of Zimbabwe, Harare, Zimbabwe; 2 Division of Molecular Biology and Human Genetics, NRF/DST Centre of Excellence for Biomedical Tuberculosis Research, South African Medical Research Council Centre for Tuberculosis Research, Faculty of Medicine and Health Sciences, Stellenbosch University, Cape Town, South Africa; 3 Department of Geography and Environmental Science, University of Zimbabwe, Harare, Zimbabwe; 4 African Institute of Biomedical Science & Technology Wilkins Hospital, Cnr J.Tongogara and R. Tangwena, Harare, Zimbabwe; 5 Department of Health, Harare City Council, Harare, Zimbabwe; Faculty of Science, Ain Shams University (ASU), EGYPT

## Abstract

**Introduction:**

The contribution of high tuberculosis (TB) transmission pockets in propagating area-wide transmission has not been adequately described in Zimbabwe. This study aimed to describe the presence of hotspot transmission of TB cases in Harare city from 2011 to 2012 using geospatial techniques.

**Methods:**

Anonymised TB patient data stored in an electronic database at Harare City Health department was analysed using geospatial methods. Confirmed TB cases were mapped using geographic information system (GIS). Global Moran’s I and Anselin Local Moran’s I (LISA) were used to assess clustering and the local Getis-Ord G_i_* was used to estimate hotspot phenomenon of TB cases in Harare City for the period between 2011 and 2012.

**Results:**

A total of 12,702 TB cases were accessed and mapped on the Harare City map. In both 2011 and 2012, ninety (90%) of cases were new and had a high human immunodeficiency virus (HIV)/TB co-infection rate of 72% across all suburbs. Tuberculosis prevalence was highest in the Southern district in both 2011 and 2012. There were pockets of spatial distribution of TB prevalence across West South West, Southern, Western, South Western and Eastern health districts. TB hot spot occurrence was restricted to the West South West, parts of South Western, Western health districts. West South West district had an increased peri-urban population with inadequate social services including health facilities. These conditions were conducive for increased intensity of TB occurrence, a probable indication of high transmission especially in the presence of high HIV co-infection.

**Conclusions and recommendations:**

Increased TB transmission was limited to a health district with high informal internal migrants with limited health services in Harare City. To minimise spread of TB into greater Harare, there is need to improve access to TB services in the peri-urban areas.

## Introduction

Tuberculosis (TB) is a respiratory infectious disease transmitted through inhalation of *Mycobacterium tuberculosis* (*Mtb*) in aerosolized droplet nuclei [1]. High population density, human immunodeficiency virus (HIV) infection, poverty, air pollution, population displacements and malnutrition increases the risk of successful TB transmission [2–4] Host characteristics like infectiousness of the infected, number of susceptible individuals, frequency of interaction and social mixing patterns plus virulence of the pathogen promote successful transmission of *Mtb* [5].

In 2017, the Africa region contributed 30% of the 30 high TB/HIV and multidrug resistant TB (MDR-TB) countries worldwide [6]. Five of these countries were from Southern Africa, a region described as the epicentre of HIV and TB infection, with high migration and extreme poverty [6–8] Zimbabwe is one of the five high TB, HIV and MDR-TB burden countries from Southern Africa with a TB/HIV co-infection rate of 68% and TB incidence of 242/100,000 in 2017 [6] Despite a falling national TB incidence, the country failed to meet the Millennium Development Goal (MDG) targets of halving the TB incidence by 50% in 2017. In line with international best practice, the Zimbabwe National TB Control Program’s (NTP)’s guidelines recommends ambulatory TB care for both sputum smear positive and smear negative TB patients [9].

In Zimbabwe, the two largest metropolitan cities, Harare and Bulawayo, contributed an estimated 30% of the national TB case load from 2008 to 2011 [10]. Health infrastructure in the two metropolitan cities is well established with well functional health facilities, good road and transport infrastructure and available free TB services to the patients. Causes of the persistently high TB case load have not been fully described besides the known effects of poverty and socioeconomic challenges affecting the general population since the early 2000. The socioeconomic challenges created social instability resulting in high rural to urban migration. For Harare and Bulawayo, the increased populations was against non-expanding social services, thus increasing the risk of TB transmission from overcrowding and poverty [11]. A national program implemented in 2008 to decongest urban areas created new peri-urban settlements where there were limited health care and other social services. This internal movement of people firstly from rural areas into major urban areas in search of livelihood and later into peri-urban areas to decongest the city, may have changed the city-wide epidemiology of TB and other poverty related diseases as evidenced by the frequent outbreaks of cholera [12,13]. Pockets of high TB transmission have been known to propagate generalized TB epidemics in urban settings [14].

Methods to estimate presence of TB hotspot transmission have been limited to either use of geographical information systems (GIS) or molecular techniques or in combination of the two [15]. The utility of geospatial techniques in providing critical planning information for TB treatment and care services has been described elsewhere but limited to high income countries [16]. Few studies have used geospatial techniques to estimate TB transmission pathways and distribution of MDR-TB clusters [15]. The aim of this study was to describe the presence of high clustering of TB cases, an indication of potential high transmission zones in Harare City using geospatial techniques for the period 2011 to 2012. To our knowledge, no studies had been done to describe the spatial pattern of TB in Harare City.

## Materials and methods

### Setting

Harare city had an estimated total population of about two (2) million in 2012, with a good road network facilitating movement of residents between the twelve (12) health districts. Each health district has several suburbs. Provision of primary health care and maternity care services are through a network of 12 polyclinics, 39 clinics and two infectious disease referral hospitals. Sputum smear microscopy was the TB diagnostic method of choice with Gene Xpert technology being prioritized for high risk drug resistant TB patients only. Culture and drug sensitivity testing for patients with first line TB treatment failure, retreatment, rifampicin mono-resistant on Gene Xpert and other high-risk drug resistant TB patients were outsourced to the National Microbiology Reference Laboratory (NMRL) at Harare Central hospital, a tertiary government referral hospital. At the end of TB treatment, all patients within the catchment area of the two referral hospitals were discharged through the respective referral hospitals for treatment outcome determination. The South west south district was a farm converted to residential area to accommodate people who had been displaced through operation restore order. It did not have social facilities including a clinic of its own.

### Sampling and sample size

Routinely collected TB diagnosis and treatment data for Harare city is stored in an Epi Info based electronic database. ll confirmed TB patient records in Harare City stored in the Epi Info database for the period 2008–2012 were retrieved. Data for 2011 to 2012 was used for this analysis because data for 2008 to 2010 was incomplete, missing patient address. Data on the physical address of the patient, name of clinic providing directly observed treatment short course (DOTS), gender, age, HIV status, treatment outcome and type of TB patient, was abstracted from the database. Population projections for Harare City was obtained from the health information unit and used for estimating district specific TB prevalence by year. Patient records with incomplete physical address and other demographic characteristics were followed up to the local clinic and used the clinic DOTS registers to retrieve the data.

### Data management

#### Demographic characteristics

Microsoft excel software was used to calculate district specific TB notification for 2011–2012. District specific populations for 2011 were extrapolated from the 2002 national census using estimated growth rate and for 2012, we used the national census report. [17] Stata version 12 was used to calculate district specific prevalence, using the number of cases reported per district and the estimated district population, frequency tables of HIV status, age and sex by year and district. This provided information on whether there were any obvious differences in factors that may have affected geographical clustering of cases and TB transmission patterns.

### Location data collection

Using the Epi Info electronic register, the TB patients’ physical address was used to get the household location. Patients’ physical location had their coordinates taken using a global positioning system receiver (GPS). We accepted a measurement error of ±3 metres. For residential areas where there was a logical chronology of physical addresses only the first and last ten household numbers had their coordinates taken and the remainder o fthe households, we used Google Earth to digitalise them. Houses that were not on google earth were mapped using the GPS receiver through field visits. The point locations were then entered in a spreadsheet and converted into .*csv* for use in a GIS environment. The points were mapped in a GIS environment using ArcMap to visualise the TB cases.

### Data analyses

#### Estimation of spatial clustering

Prevalence of TB per health district was calculated based on the district TB case notification and estimated district population. District specific HIV prevalence was calculated from the total HIV positive divided by the total tested for HIV. Before detecting the pattern of TB prevalence in Harare over the two years, there was need to test for the presence and nature of TB occurrence. We tested whether the occurrence of TB had a tendency to form a systematic pattern than would be under the hypothesis of randomness i.e., spatial autocorrelation (or dependency). To achieve this, we applied both local and global measures of spatial autocorrelation. To achieve this, the Global Moran’s Indices was applied. The global Moran’s Index was applied to confirm presence of non-random distribution of TB cases in all suburbs in Harare. The presence of spatial autocorrelation in terms of TB disease estimated the levels of TB clustering, which is indicative of hierarchical expansionary spread in urban areas and across the health districts. With an index ranging from -1 to +1, a score of zero indicated no spatial autocorrelation and a positive value indicated spatial clustering of TB cases across suburbs. A negative value show that neighbouring suburbs were characterised by dissimilar TB cases [18]. In addition to the Global Moran’s I, there was need to understand the type of spatial correlation in the distribution of TB cases by health districts. The Anselin Local Moran’s I (LISA) was applied to assesses the influence of individual TB cases within surrounding health districts on other districts [19]. The results of LISA classified health districts into clusters of high TB cases next to high TB cases i.e., high-high, (HH), (low TB cases next to low cases, (LL), “coldspots” and spatial outliers (high amongst low, (HL) or vice versa, (LH). LISA was computed using the following formula [20]:

$$Iᵢ=\frac{\sum_{j=1}^{n}\;W_{ij}(x_{i}-\overset{-}{x})(x_{j}-\overset{-}{x})}{\frac{1}{n}\sum_{i=1}^{n}\left(xᵢ-\overset{-}{x}\right)²},i\neq j$$

where n was the number of suburbs, x_*i*_ and x_*j*_ were the positive TB cases of suburb *i* and *j*, respectively; $\overset{-}{x}$ is the average of the reported TB cases of all suburbs in Harare, and *W*_*ij*_ was the spatial weight matrix corresponding to the suburb pair *i* and *j*. Suburbs with high TB cases were classified as hot spots, while the ones with low TB cases were classified as cold spots. Spatial outlier represented the location where there was a mixture of high and low TB cases in neighbouring suburbs. The computation of LISA resulted in five scenarios of results: High-High, Low-Low, Low-High, High-Low, and Not Significant.

#### Estimation of intensity of TB spatial distribution

The local Getis-Ord G_i_* statistic, a hotspot analysis function in ArcGIS (Ver.10.2, ESRI Inc., CA, USA), was used to test for the intensity of incident TB cases, a measure of hotspots or cold spot occurrence TB in Harare [21,22]. The calculated ratio of the local sum of the TB cases in the vicinity of a suburb based on a five (5) metre threshold was compared to the total sum of all the TB cases in each health district to estimate intensity of incident TB cases. The intensity of incident TB cases was used to estimate transmission within the health districts. The statistical significance of a Z-score for each suburb was quantified through the presence of hotspots and coldspots of TB incidence, relative to the hypothesis of spatial randomness [23]. The Getis-Ord G_i_* calculates a Z-score where a significant positive Z score (Gi*) indicated hotspot phenomenon. A negative Z-score showed cod hotspot [21,22]. Suburbs with a Z scores > 1.96 at 99% confidence level (p < 0.01) were categorised as hotspots of TB. Likewise, suburbs with a Z-score of <−1.96 indicated coldspots TB clustering.

#### Ethical consideration

This study was approved by the relevant institutional review boards, Medical Research council of Zimbabwe (MRCZ), number MRCZ/A/1830 and Stellenbosch institutional review board, S16/06/106. De-identified and routinely collected secondary clinical patient data was used. This study was part of a PhD study on migration and TB transmission in Zimbabwe. Ethics permission was on the full protocol including this spatial epidemiology study. Permission to use historical data was provided by Harare City health department and informed consent was waivered by the ethics review committee on the basis that no patient contact was used to collect data and identifiable patient record was removed except the full patient address that was used for GIS. After GIS mapping from the peri-urban settlements where there was no identifiable chronological order of the households, full address information was removed before data analysis.

## Results

### Background characteristics of cases notified by Harare City, 2011–2012

**Fig 1:**
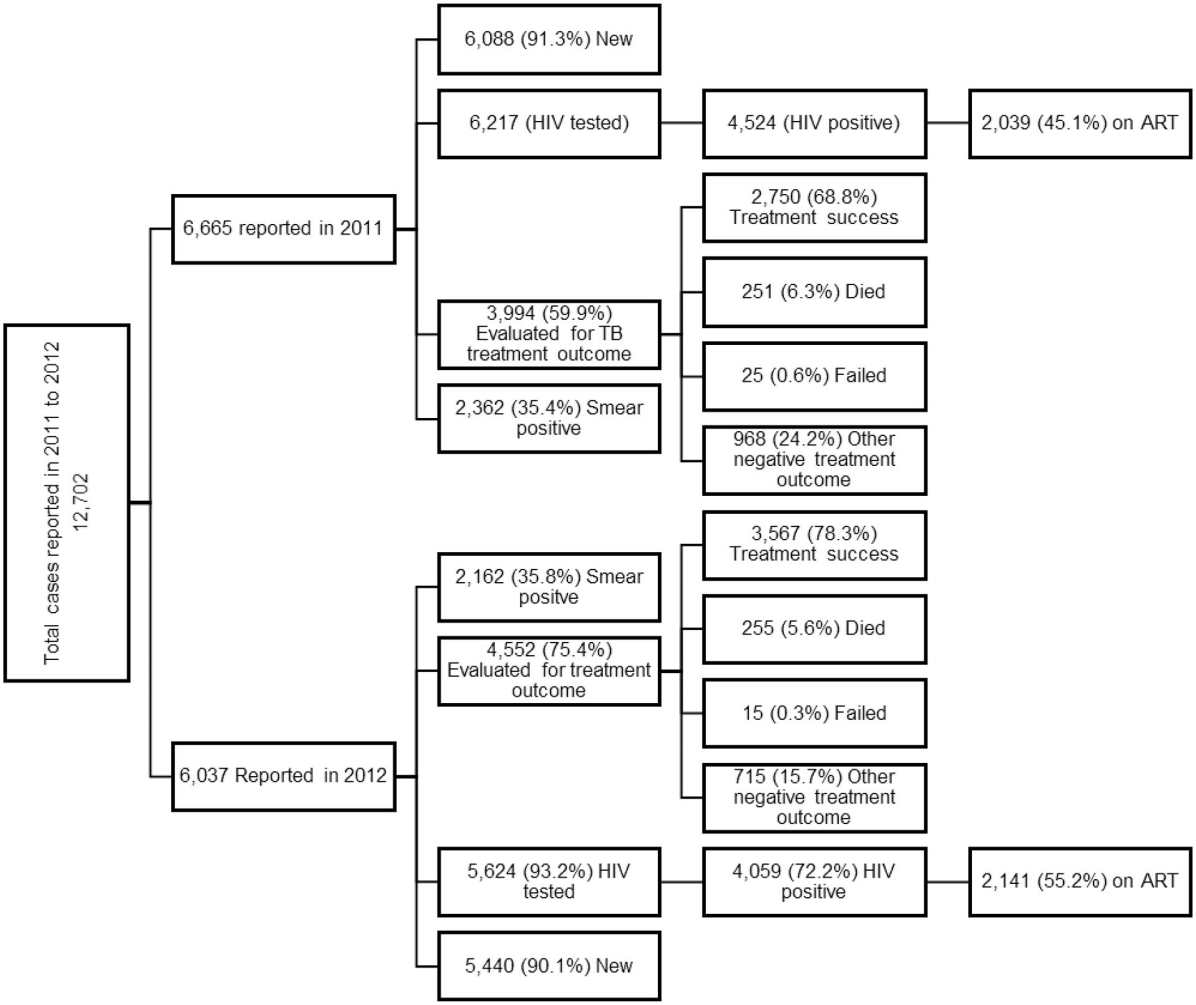
Characteristics of tuberculosis patients receiving treatment in Harare City, 2011–2012. The figure summarises the total TB cases included in the study disaggregated by year of treatment, TB patient type, HIV testing and antiretroviral therapy (ART) status and treatment outcome for 2011 and 2012, Harare City.

Harare City notified a total of 12,702 TB patients between 2011 and 2012 (Fig 1). Ninety (90%) of all TB cases were new and 4,524 (35.6%) of the patients had sputum smear positive TB. Percentage HIV prevalence ranged between 65% to 77% in Central and North Western districts respectively in 2011 (Table 1). In 2012, HIV prevalence ranged between 68% to 76% in South eastern and Southern districts respectively. Across health districts, the HIV/TB co-infection was similar. Prevalence of TB was lowest in Central district and highest in Southern district for both 2011 and 2012, 158/100,000 population to 651/100,000 population in 2011 and 190/100,000 population and 648/100,000 in 2012 respectively (Table 2).

**Table 1:** TB and HIV prevalence by health district, Harare City, 2011–2012.

Year	2011				2012			
District	# of TB cases	Estimated Total Population	TB Prev/ 100,000	HIV prev (%)	# of TB cases	Estimated district population	TB prev/ 100,000	% HIV prev
Central	91	57640	158	65	111	58281	190	73
Eastern District	686	145988	470	74	540	147612	366	70
Nothern District	300	103395	290	76	265	104545	253	73
North Western District	783	221196	354	77	737	223656	330	74
Southern District	1,073	164748	651	75	1,079	166580	648	76
South Eastern District	180	44121	408	73	161	44612	361	68
South Western	880	197012	447	76	756	199203	380	71
West	890	247597	359	73	859	250351	343	73
WSW	1,082	287197	377	70	988	290391	340	72

**Table 2:** Demographic characteristics of TB patients receiving treatment in Harare City, 2011–2012.

Variable	2011 N (%)	2012 N (%)	P-value
**Age**			
<5 years	290 (4.4)	252 (4.2)	0.023
5–10 years	169 (2.5)	146 (2.4)	0.191
**11–19 years**	**320 (4.8)**	**332 (5.5)**	**<0.001**
20–44 years	4468 (67.0)	4005 (66.3)	0.403
45+ years	1418 (21.3)	1302 (21.6)	0.681
Total	6665 (100)	6037 (100)	
**Gender**			
Male	3755 (56.3)	2586 (57.2)	0.307
Female	2910 (43.7)	3451 (42.8)	
Total	6665 (100)	6037 (100)	

The age-groups 20–44 years old was the worst affected by TB, accounting for 67% of cases in 2011 and 66% in 2012 respectively, followed by the age group >45 years old that averaged 21% for both 2011 and 2012 (Table 1). In 2012, children between 11–19 years were significantly more likely to have TB compared to 2011, 4.8% vs 5.5%, p<0.001. There were no differences in age-group and gender distribution by district. More males had TB (average 56.7%) than females. The proportion of sputum not done was relatively small for both 2011 and 2012, (14.4% and 8.7% respectively). Across all districts, HIV testing was high (>90%), and the HIV /TB co-infection rates averaged 72%, for both 2011 and 2012 across all districts. A combined 50% of all TB/HIV co-infected patients received antiretroviral therapy (ART), 4,280 out of the total HIV positive of 8,588. Treatment success rate was below the WHO recommended of 85%, 79.3% and 68.8% for 2011 and 2012 respectively. The number of TB patients completing treatment but not evaluated was high, 24.3% in 2011 and 15.7% in 2012 but mortality was below 10%.

### Spatial distribution of tuberculosis prevalence rates by district, Harare City, 2011–2012

**Fig 2:**
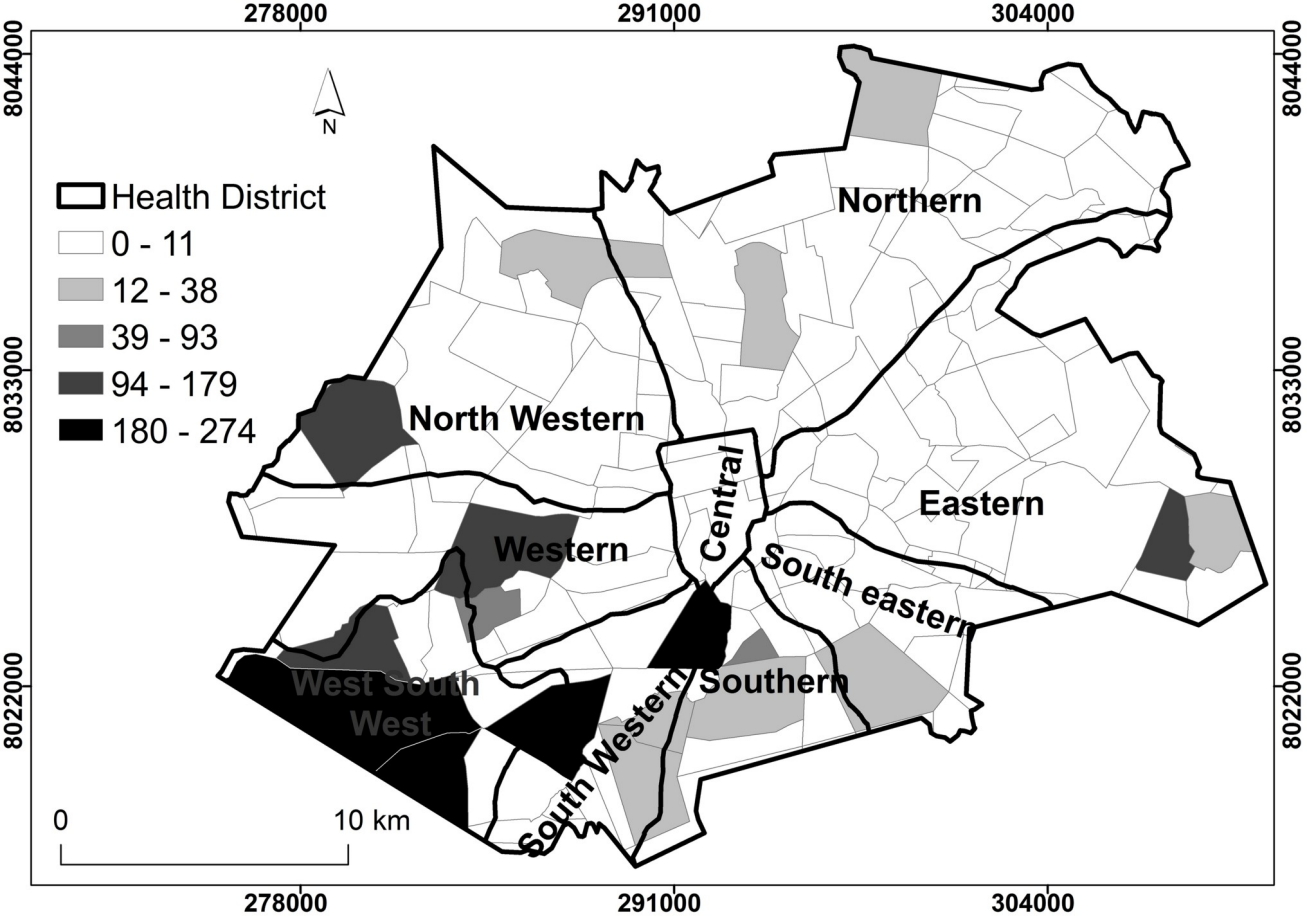
Spatial distribution of TB prevalence rates by district, Harare City, 2011. Show the spatial distribution of TB prevalence rates by district. Clear suburbs and districts show that TB cases were not related to neighbouring districts. Shaded districts showed relatedness. The intensity of the shade reflected the number of TB cases per district related to neighbouring districts.

**Fig 3:**
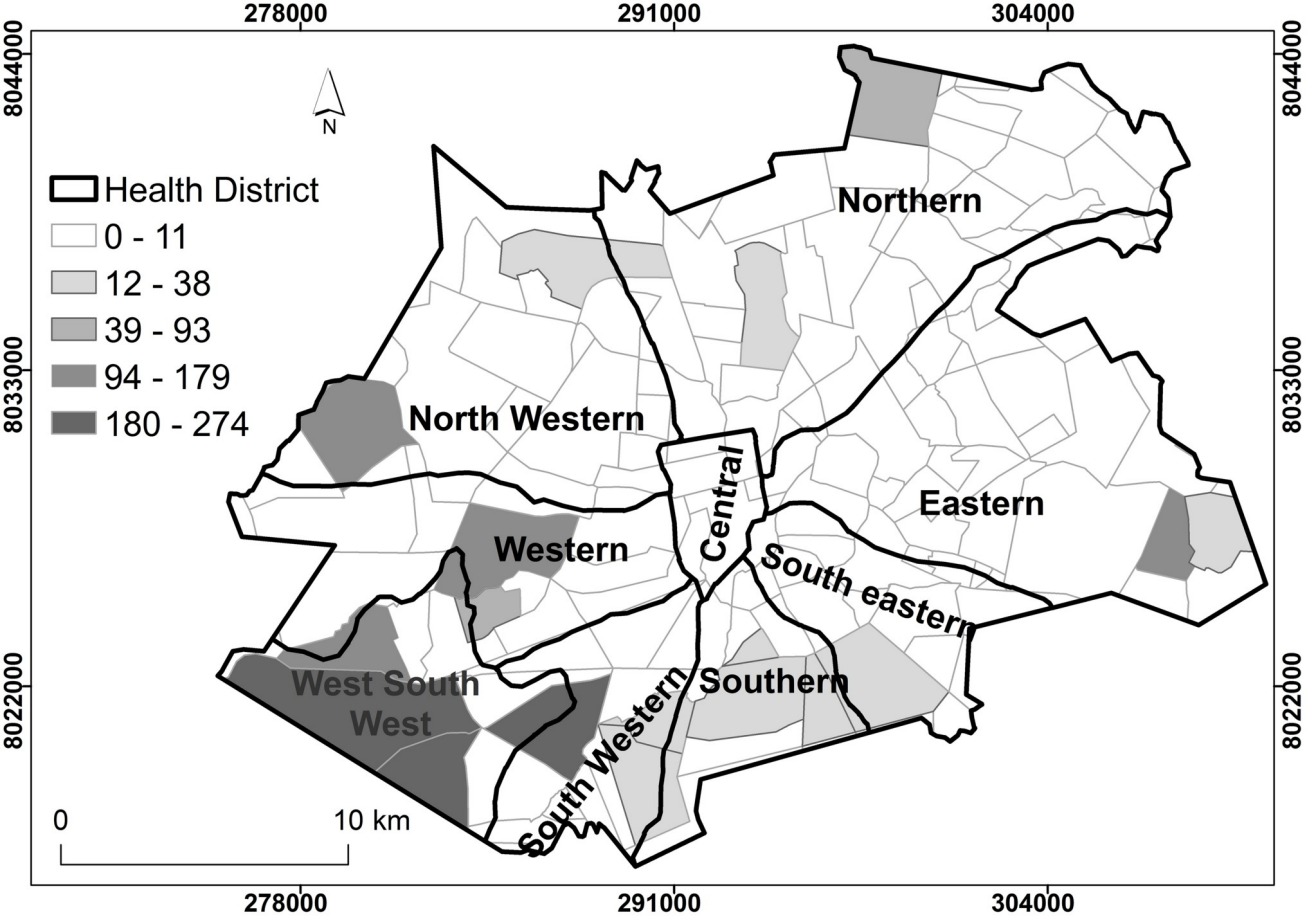
Spatial distribution of TB prevalence rates by district, Harare City, 2012. Show the spatial distribution of TB prevalence rates by district. Clear suburbs and districts show that TB cases were not related to neighbouring districts. Shaded districts showed relatedness. The intensity of the shade reflected the number of TB cases per district related to neighbouring districts.

Figs 2 and 3 show the spatial distribution of TB prevalence by health district for Harare City. In both 2011 and 2012 most of the health districts were characterised by suburbs with low (0–11) spatial distribution of TB prevalence, that is, majority of the districts had TB cases that were unrelated to each other. Global spatial autocorrelation for parts of Northern, Eastern, north western, western, south western and south west south, located in the periphery of the greater Harare showed presence of positive spatial clustering of TB cases for both 2011 and 2012 (p-value < 0.05). The clustered distribution of TB cases in the parts of Harare’s peripheral districts indicated the non-random occurrence of the TB cases in the central business districts. Suburbs in the Southern and Western areas of the city had the highest TB prevalence especially the West South West (WSW), South Western and Eastern districts that were related to each other.

### Local spatial clustering of TB cases in Harare City

**Fig 4:**
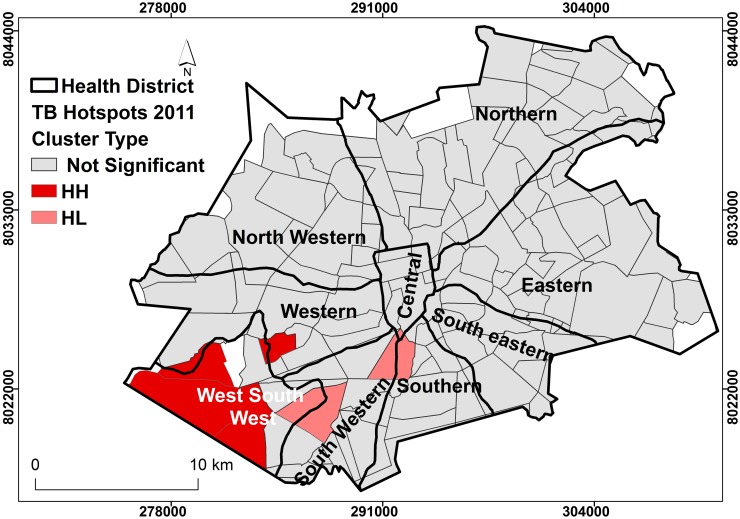
Clustering of TB cases by district, Harare City, 2011. Show the clustering of cases within district. Suburbs with deep shade represent increased occurrence of TB cases that are related to other cases within the same district, (clustering). Districts with clear colour show no clustering at all.

**Fig 5:**
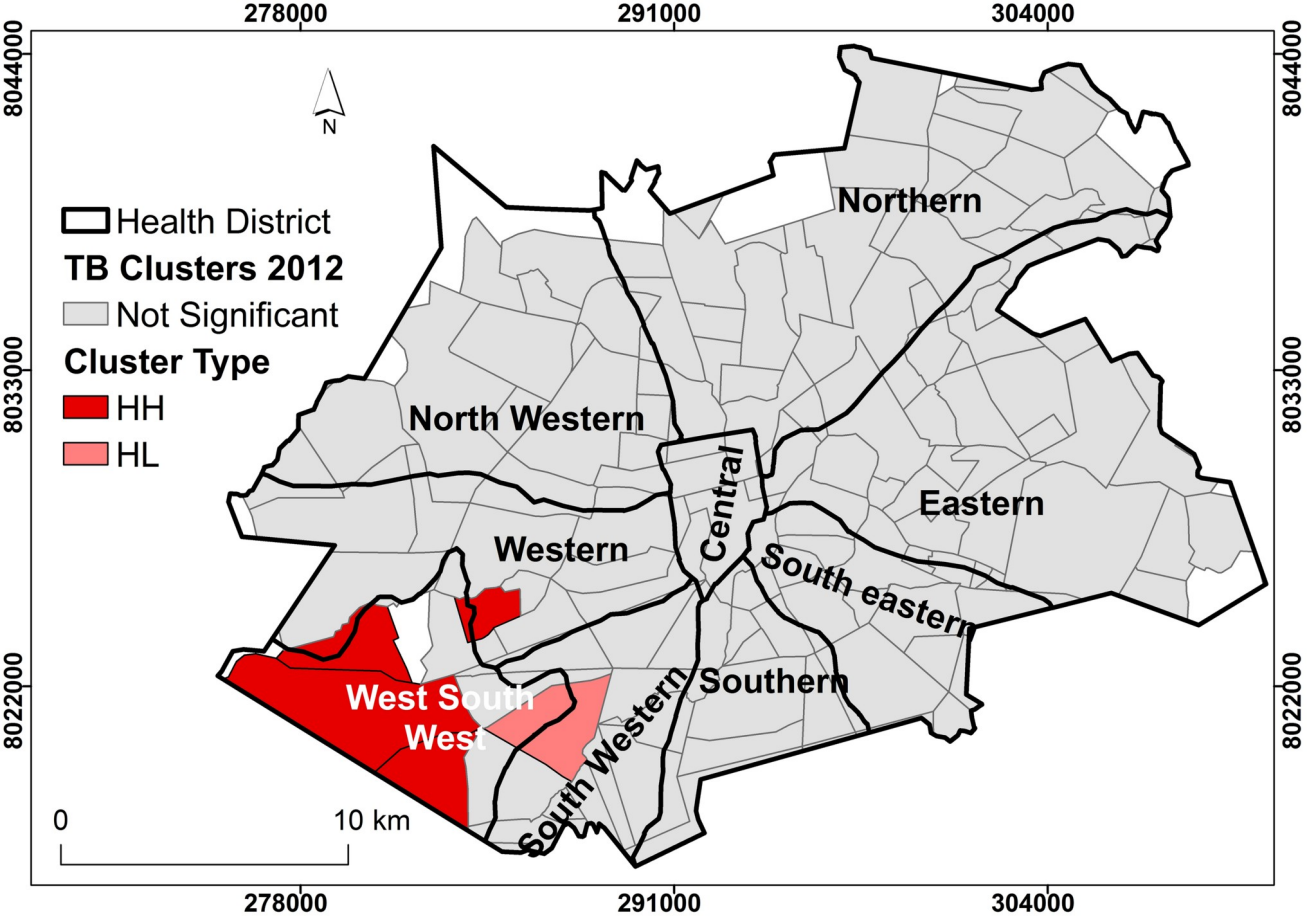
Clustering of TB cases by district, Harare City, 2012. Show the clustering of cases within district. Suburbs with deep shade represent increased occurrence of TB cases that are related to other cases within the same district, (clustering). Districts with clear colour show no clustering at all.

The results of Anselin’s Local Moran’s I analysis estimated the presence of clustering of TB cases within specific suburbs and districts. Presence of clustering crudely measured the presence of active transmission. The Southern and Western suburbs had TB cases that were more likely to be clustered than the rest of Harare City (Figs 4 and 5). In 2011, there were more suburbs and districts with clustering of TB cases than 2012. Interesting to note that despite the high incidence of TB cases in the Eastern district, the cases were more dispersed and seemed to demonstrate random occurrence for both 2011 and 2012.

### Intensity of TB case incidence (Hot spot)

**Fig 6:**
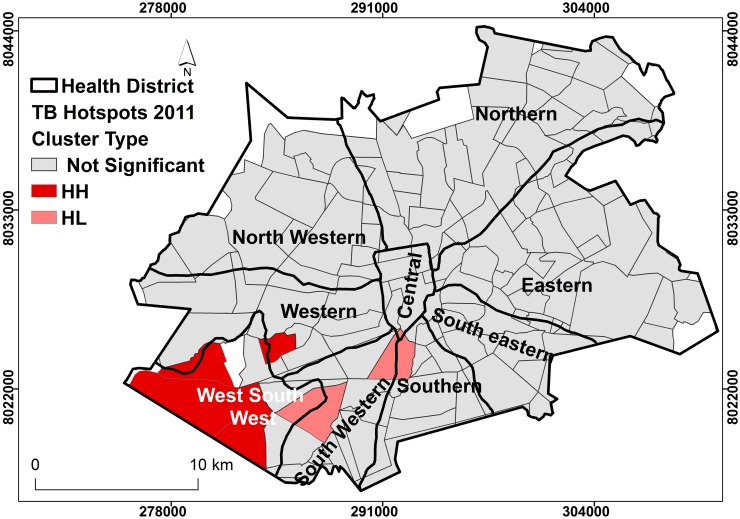
Hotspot pattern, Harare City 2011. **TB** Light highlighted areas (grey) had z-score of <1.96, indicating absence of hotspot pattern and less likely to have active TB transmission. Dense highlighted areas (red) had z-score >1.96, indicating presence of hotspot pattern and more likely to have active TB transmission.

**Fig 7:**
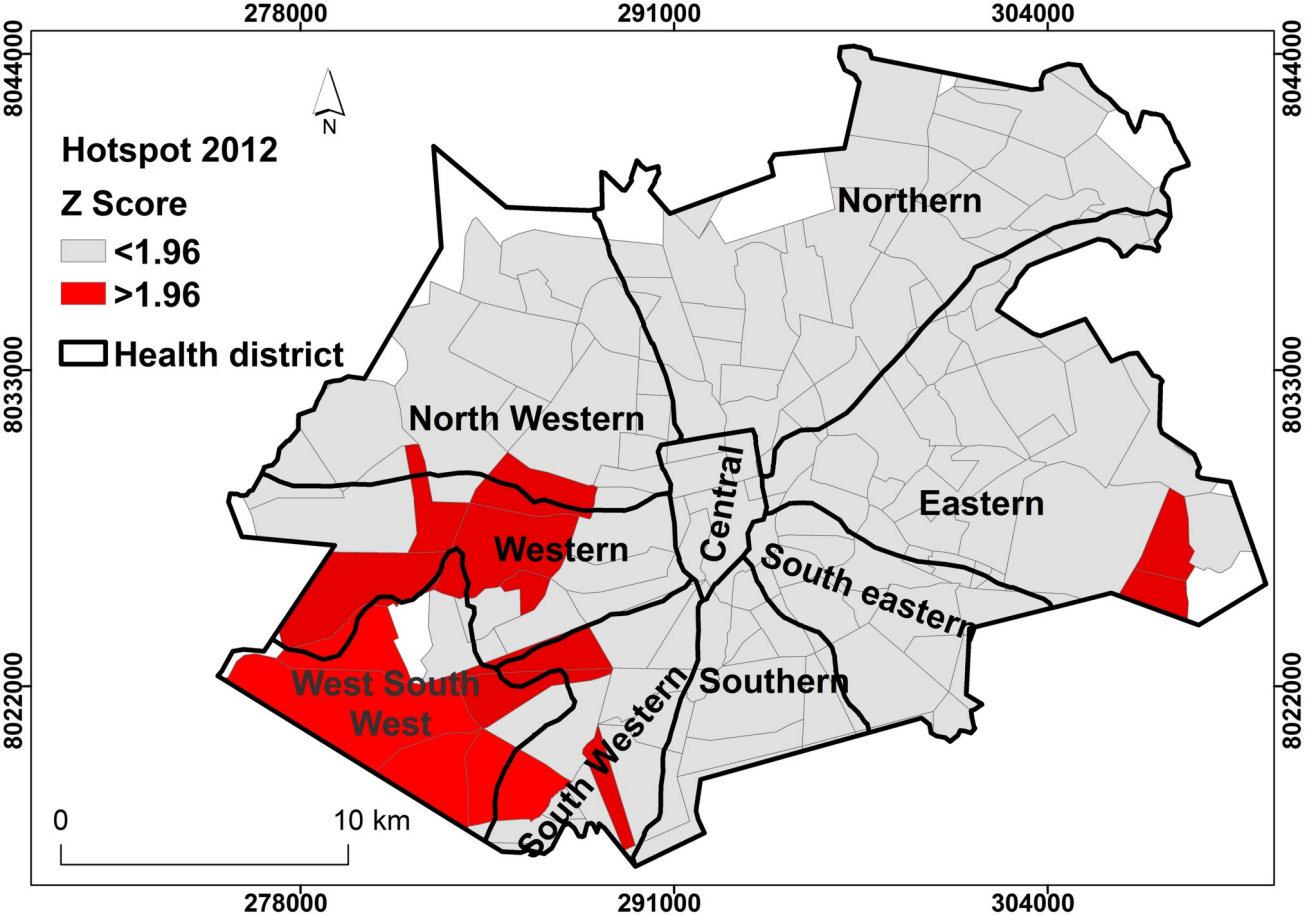
TB Hotspot pattern, Harare City, 2012. Light highlighted areas (grey) had z-score of <1.96, indicating absence of hotspot pattern and less likely to have active TB transmission. Dense highlighted areas (red) had z-score >1.96, indicating presence of hotspot pattern and more likely to have active TB transmission.

Figs 6 and 7 show results of local Getis-Ord G_i_* statistic analysis. The results showed that the intensity of spatial distribution of TB cases, hotspot phenomenon, was highest in the west-south-west district of Harare city. This local pocket of TB incidence was similar for both 2011 and 2012. In 2011, some parts of the North Western, western and south western districts showed high intensity of TB spatial distribution. However, in 2012, some parts of the Eastern and south western districts showed some areas of increased intensity of TB occurrence. Within the West-South-West district, suburbs with the highest intensity of TB incidence were Budiriro, Glenview, Glen Norah and Mbare suburbs.

## Discussion

Our results showed that the TB epidemiology in Harare City was mainly characterized by new patients, affecting the most economically active age group of 20–44 years, had a high TB and HIV co-infection rate of 72% and seemed to have been driven by small areas of intense TB transmission.

Previous studies have shown that the economically active 25–49 years age-group and male population had the highest risk of TB and HIV infection [24]. In Harare, proportion of the working population aged 25–49 was the highest due to the presence of employment opportunities [25]. This may explain the high case load contributed by Harare City to the national burden. The high proportion of new TB cases may indicate that transmission could be the most common mode of acquiring TB disease in Harare as described in studies from South Africa and Zimbabwe [5,10]. In a country with adequate funding from the partners and Government and no reported shortage of anti-TB medicines, consumables and supplies, the observed high prevalence of new TB cases suggesting continuous transmission require a more focused investigations to understand the drivers of transmission. The high HIV co-infection across all districts maybe driving the TB epidemic. However, despite a similar average HIV prevalence, there were differences in TB prevalence, (cases per capita) by district and also spatial distribution suggesting that HIV alone was not the cause of the observed TB distribution as reported from a study from Ethiopia [26].

Findings showing an increased risk of TB among the 11–19 years age-group were unusual in our study. There are two possible explanations for these findings. First, the prevention of mother to child transmission (PMTCT) of HIV in Zimbabwe programme had significantly reduced mother to child transmission of HIV in Zimbabwe and those HIV positive children were living longer due to high coverage of antiretroviral therapy (ART) [27] Therefore an accumulation of high risk HIV positive young people in an environment of high TB transmission may have resulted in the increased TB transmission in this age group. Secondly, a study in Cape Town, South Africa showed that adolescent population was more likely to contract TB whilst away from home, mainly at school [28] This could explain the relatively high prevalence of TB in the school going 11–19 years age-group in our study.

Although the TB prevalence (cases per population) showed that the Southern region had the highest TB prevalence, the global Moran’s I test showed that the Southern region TB cases were not highly related to any of the neighbouring districts. This shows the utility of analysing epidemiological data in terms of space and time rather than rely on routinely collected surveillance data only. In the Southern district are three health facilities, two clinics and one infectious disease referral hospital. Also, the country’s main public transport terminus is located in the Southern district. We hypothesized that the increased prevalence of unrelated TB cases could have been due to people accessing TB services in health facilities from the Southern district but staying outside the district. The contribution of this internal movement for health care services to the TB epidemic remains an area of research need in Zimbabwe as some studies from South Africa have reported TB transmission associated with the transport sector. [29]

Geospatial analysis of TB cases in Harare city showed that the occurrence was not random. The suburbs with high TB prevalence and showing high transmission intensity were similar in that they were both located in the periphery of the city. In addition, they were providing services to a peri-urban population that had been created by the high rural to urban migration and efforts to decongest Harare city. The worst affected districts were the Southern, West South West and Eastern districts. Studies describing geographic clustering of TB cases found that the most common risk factors for clustering were low socioeconomic status, homelessness, poor housing and low education attainment [30,31] Successful transmission of TB require the presence of infectious cases that interact with susceptible uninfected individuals for a longer period of time [32] Conditions that affect early TB diagnosis, early treatment and access to social services therefore promote ongoing transmission of TB. West South West and Southern districts had pockets of peri-urban populations that had no access to health care services and were relying on services outside where they lived. Studies from China and Ethiopia also reported that low access to health care services was associated with increased number of TB cases. [26,33].

The Eastern district showed presence of clustering, an indication of relatedness of the TB cases and maybe ongoing transmission. However, there was no evidence of increased intensity of relatedness as observed in West South West district. A few reasons could explain this observation. The Eastern district was next to a rural and farming district that had adequate health facilities, with no overcrowding and relatively less poor. The rural and farming community next to the Eastern district had access to district hospital and several rural health centres servicing the communities. In addition, several small farms next to the Eastern district provided economic activity for the populations and reduced the need to travel towards the Eastern and central districts.

Assessment of TB transmission had used TB notification data in children, population based surveys using interferon gamma release assays, geospatial techniques and more recently whole genome sequencing [32] Molecular epidemiological studies have been criticized for failing to distinguish changes in transmission intensity. The use of geospatial techniques in the African continent have been limited due to software costs and access to spatial data although they provide evidence on changes in transmission intensity [30]. Optimal methods of assessing TB transmission would be to combine molecular methods with geospatial techniques. [15] In our study, we demonstrated the utility of geospatial techniques to provide critical information for public health planning in TB programming.

Our findings from the first geospatial study of TB epidemiology in Harare brought out several issues critical for targeted TB prevention and control. Firstly, the results have demonstrated that in the absence of adequate public health services, social diseases like TB tend to occur more commonly. The increased contact or social mixing resulting from increased movement in search of social services and livelihood, may have been propagating TB transmission in Harare city and the general population [34]. This may explain why Harare city, has been contributing the highest TB case load in Zimbabwe. The city is one of the densely populated, contributing about 16.3% of the total population, hence facilitating increased contact between TB susceptible and infected persons [25].

Secondly, despite the decline in the national TB incidence in Zimbabwe, pockets of high TB transmission like Harare city will continue to propagate the disease at national level. Similar findings from metropolitan cities have attributed pockets of increased TB transmission, hotspots, to the continued generalized TB epidemics [14]. The mechanism of spread is believed to be increased social interaction as populations move in search of social services including health care [4,35]. Identifying areas of increased TB transmission and plan interventions accordingly is critical for controlling the TB epidemic in Harare City. Also critical is the recognition of non-health sector developmental issues that may affect distribution TB disease.

Thirdly, our results show that use of geographical information systems (GIS) provides important information on social and environmental characteristics of TB transmission in addition to the known epidemiological characteristics [5]. With most of the available evidence of spatial epidemiology of TB have been limited to describing the TB case load in terms of prevalence over time, the use of inferential GIS statistics like the Anselin’s Local Moran’s I and Getis Gi statistics provided additional information on TB transmission dynamics [15,21,30]. This information is important in defining areas that require targeted health and developmental interventions.

Findings from this study may have been affected by use of retrospective secondary data. This limited the time duration of follow up, as the years 2008–2010 were excluded due to missing variables on physical address. Follow up of contacts to assess the proportion of secondary cases and confirm the behavioral characteristics of TB patients reported in Harare city was not possible. An anthropological study to adequately characterize and understand the TB epidemic in Harare City is recommended [36–38]. It was not possible to estimate the direction of the hotspot because of the short two years duration of follow up.

Despite the above limitations, we conclude that reduced access to public health services in Harare’s peri-urban areas was contributing to the ongoing TB epidemic in Zimbabwe. The authors recommend that Harare City council provide public health services to peri-urban areas to interrupt TB transmission. This could be done through establishing health posts with capacity to screen and diagnose TB using Gene Xpert as outlined by the current national health strategic plan. In addition, Harare City health should strengthen capacity of the Western, Southern and Eastern districts to provide early TB case detection and treatment to interrupt TB transmission. Working with other government sector ministries, Harare City council should ensure economic activities are available for the newly established peri-urban settlements. This would reduce population movements in the inner city and thereby reducing potential for spreading infectious diseases like TB.
